# Structure and evolutionary implications of the earliest (Sinemurian, Early Jurassic) dinosaur eggs and eggshells

**DOI:** 10.1038/s41598-019-40604-8

**Published:** 2019-03-14

**Authors:** Koen Stein, Edina Prondvai, Timothy Huang, Jean-Marc Baele, P. Martin Sander, Robert Reisz

**Affiliations:** 1 Earth System Science - AMGC, Vrije Universiteit Brussel, Pleinlaan 2, 1050 Brussels, Belgium; 2 Royal Belgian Institute of Natural Sciences, Directorate ‘Earth and History of Life’, Rue Vautier 29, 1000 Brussels, Belgium; 3 Evolutionary Morphology of Vertebrates, Ghent University, K.L. Ledeganckstraat 35, 9000 Gent, Belgium; 4 MTA-ELTE Lendület Dinosaur Research Group, Eötvös Loránd University, Pázmány P. s. 1/C, 1117 Budapest, Hungary; 5 International Center of Future Science, and Dinosaur Evolution Research Center of Jilin University, Changchun, Jilin Province China; 6 National Chung Hsing University, Taichung, 402 Taiwan; 7 Department of Geology and Applied Geology, Faculty of Engineering, University of Mons, Place du Parc 20, 7000 Mons, Belgium; 8 Steinmann Institute of Geology, Mineralogy, and Paleontology, Division of Paleontology, University of Bonn, Nussallee 8, 53115 Bonn, Germany; 9 Natural History Museum of Los Angeles County, Dinosaur Institute, 900 Exposition Boulevard, Los Angeles, CA 90007 USA; 10 Department of Biology, University of Toronto Mississauga, Mississauga Ontario, L5L 1C6 Canada

## Abstract

One of the fossil record’s most puzzling features is the absence of preserved eggs or eggshell for the first third of the known 315 million year history of amniote evolution. Our meagre understanding of the origin and evolution of calcareous eggshell and amniotic eggs in general, is largely based on Middle Jurassic to Late Cretaceous fossils. For dinosaurs, the most parsimonious inference yields a thick, hard shelled egg, so richly represented in the Late Cretaceous fossil record. Here, we show that a thin calcareous layer (≤100 µm) with interlocking units of radiating crystals (mammillae) and a thick shell membrane already characterize the oldest known amniote eggs, belonging to three coeval, but widely distributed Early Jurassic basal sauropodomorph dinosaurs. This thin shell layer strongly contrasts with the considerably thicker calcareous shells of Late Jurassic dinosaurs. Phylogenetic analyses and their Sinemurian age indicate that the thin eggshell of basal sauropodomorphs represents a major evolutionary innovation at the base of Dinosauria and that the much thicker eggshell of sauropods, theropods, and ornithischian dinosaurs evolved independently. Advanced mineralization of amniote eggshell (≥150 µm in thickness) in general occurred not earlier than Middle Jurassic and may correspond with a global trend of increase in atmospheric oxygen.

## Introduction

**Figure 1:**
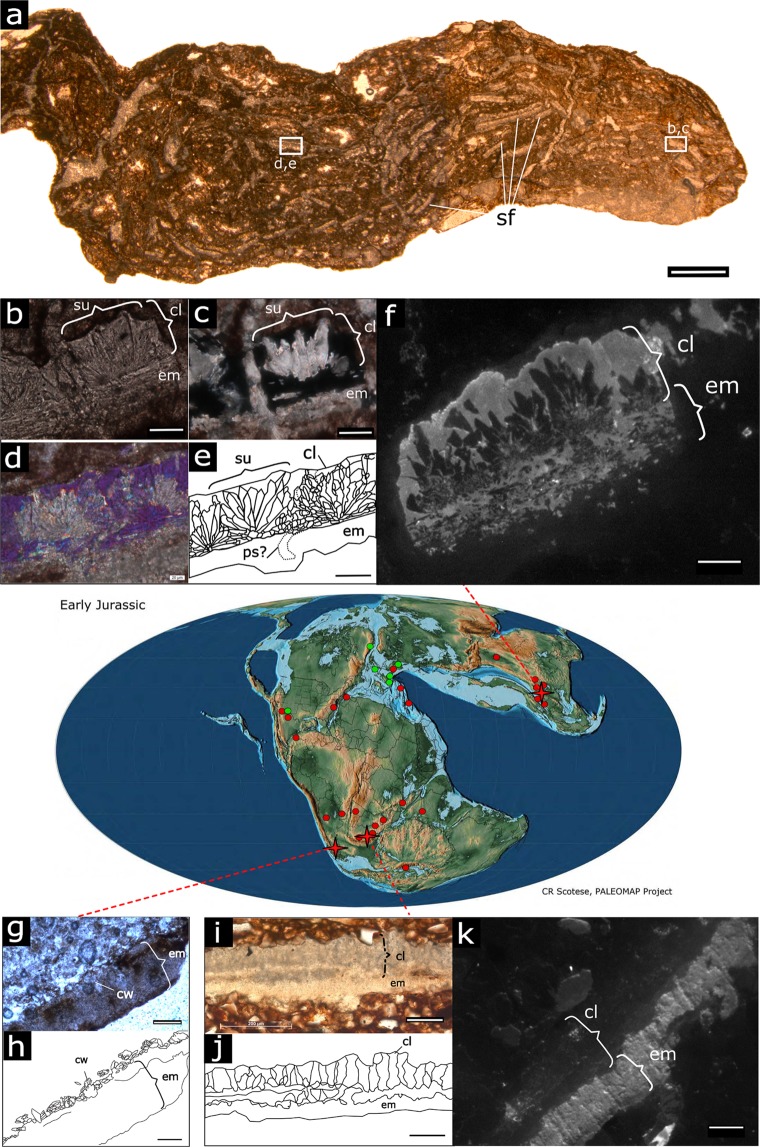
Basal sauropodomorph eggshell microstructure and their respective Sinemurian localities (crosses) among the Rhaetian (green) to Sinemurian (red) global record of sauropodomorph fossil sites (circles). (**a**–**f**), *Lufengosaurus* (Chuxiong Prefectural Museum, catalogue no. C2019 2A233), (**g**,**h**), *Mussaurus* (Instituto ‘Miguel Lillo”, Tucuman, catalogue no. PVL 5965), (**i**–**k**), *Massospondylus* (Bernard Price Institute of Palaeontology, University of Witwatersrand, catalogue no. BP/1/5254). (**a**) Section through nugget containing numerous *Lufengosaurus* eggshell fragments (plane polarized light, ppl). (**b)**, close-up (ppl) of a *Lufengosaurus* eggshell fragment, showing calcite crystals of the mammillary layer radiating from an organic core embedded in the eggshell membrane. (**c**) As in (**b**) under cross polarized light (xpl), highlighting the calcite crystals of a mammillary cone. (**d**) Different xpl view with lambda waveplate, e. line drawing of (**d**). (**f**) *Lufengosaurus* cathodoluminescence view with 880 nm filter. (**g**) *Mussaurus* eggshell, showing thick eggshell membrane, and distorted calcareous layer. (**h**) Line drawing of (**g**). (**i**) *Massospondylus* eggshell fragment (ppl), showing wedges in the calcareous layer, and a homogenous eggshell membrane. (**j**), Line drawing of (**i**). (**k**) *Massospondylus* cathodoluminescence view with 880 nm filter. Scale bars: in (**a**): 1 mm, (**b**–**f**,**k**): 50 µm, (**g**–**j)**: 100 µm. Abbreviations: cl, calcareous layer; cw, crystal wedges of calcareous layer; em, eggshell membrane; ps, pore space; su, shell unit. See also Figs S1–S3. (Map from^66^ with permission).

The origin of the amniote egg is a topic of great significance because it represents one of the major evolutionary innovations in vertebrate evolution, allowing the group to complete their invasion of the terrestrial landscape and sever their reproductive cycle from the aquatic medium^1^. However, paleontological studies of this pivotal event have been greatly hampered by the poor early record of fossil eggs^2,3^. Recent attempts to fill the gaps in fossil eggshell phylogeny still leave at least 125 million years of amniote evolution between the appearance of amniotes in the fossil record and the first appearance of preserved terrestrial eggs or eggshells^4–12^. The oldest known eggs or eggshells have been reported^7–12^ from three Sinemurian (195-192 Ma) sauropodomorph dinosaurs, *Massospondylus* from the Elliot Formation of South Africa, *Lufengosaurus* from the Lufeng Formation of Yunnan, China, and *Mussaurus* from the Laguna Colorada Formation of Argentina (Fig. 1). Within the context of their respective localities, some of these materials have been examined to a limited extent. In a study on prenatal remains of *Lufengosaurus*, some of the authors of the current study previously provided a very brief description of its eggshell, and noted its extreme thinness^12^. Other authors working on *Massospondylus* initially discarded them as crocodile eggshells^13^, and later as having a diagenetically altered microstructure^9^. *Mussaurus* eggshell has to our knowledge never been described in a formal publication. These remains are the earliest confirmed amniote eggshells recorded in the fossil record. Due to their rarity, fragmentary nature, and great geographic distance from each other, they were never studied from the perspective of the evolution of amniote eggshell. Here we aim to understand their microstructural features and try to elucidate when and how the earliest mineralized eggshells could have evolved. To accomplish our goal, we utilised petrographic sections, analytical chemistry tools and computational statistical methods (description in Materials and Methods section). This study contributes to our understanding of the evolution of rigid shelled eggs; a key trait in the evolutionary success of archosaurs.

## Results

### Eggshell structure

The calcareous layer of *Lufengosaurus* eggshells (C2019 2A233) ranges 60–90 µm in thickness. They consist of crocodile eggshell-like wedge- and crown-shaped shell units that are relatively wide compared to the calcareous layer thickness (Fig. 1a–f). Polarized light microscopy suggest that the outer surface of the eggshell is unaltered (Fig. 1f, Supplementary Information). The very thin crystalline layer (~10 µm) topping the eggshell units is phosphatic in nature (Figs S1 and S2), and looks scalloped with shallow pits and low ridges, not necessarily matching eggshell unit borders. These surface irregularities or tubercles are of such small dimensions that the surface of the calcareous layer looks smooth (Fig. S2a), and it remains unclear if they match the ornamentations seen in younger dinosaur eggshells. The bulk of the units, corresponding to the mammillary cones, is formed by a calcite radial ultrastructure (*sensu*^14^) with interlocking crystalline units (Fig. 1c–f). The patchy cathodoluminescence texture suggests some radial crystal wedges experienced recrystallization, but most of the original microstructure is conserved (Fig. S1b. No tabular structures or horizontal accretion lines can be observed. The growth centre of the units is embedded in a phosphorus-rich (Figs S1 and S2), thick fibrillar layer (60–75 µm) representing the eggshell membrane (Fig. 1e). Pore spaces are rare and difficult to discern (Fig. 1e). Due to the fragmentary nature of the materials, and because pores were not always unambiguously identifiable, it was not possible to make an estimation of pore density. However, pore distribution does not appear to be consistent with the presence of tubercles or depressions on the outer surface. A tangential section through the membrane shows clusters of crystals with flower-like arrangements (Fig. S3). The lack of a thick palisade layer and the overall thinness of the calcareous shell clearly distinguish *Lufengosaurus* eggshells from avian and other younger dinosaurian eggshells.

**Figure 2:**
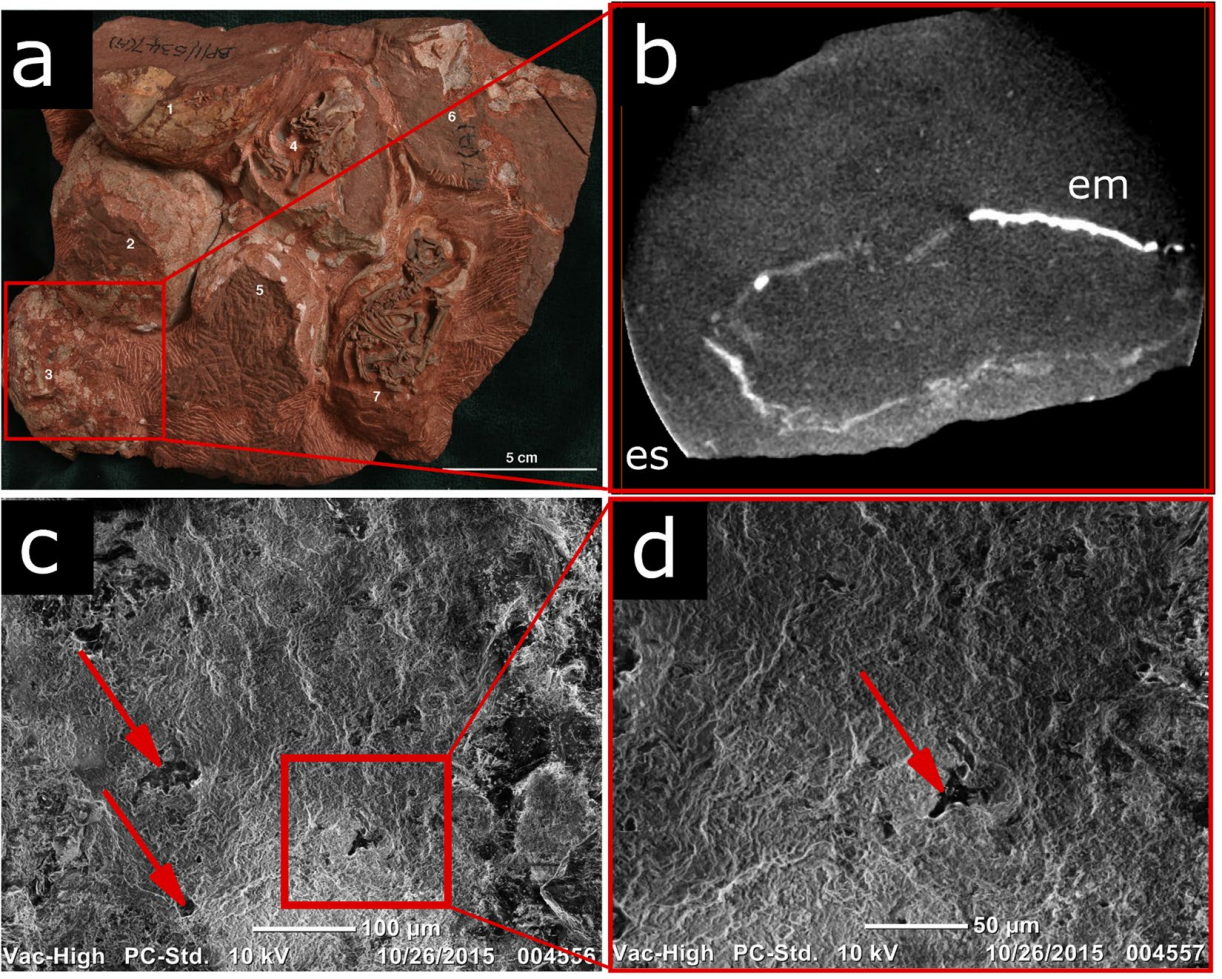
Eggshell membrane and porosity in *Massospondylus* eggs (BP/1/5347). (**a)** Nest of *Massospondylus* eggs with preserved embryos. Note the presence of numerous cracks in the eggs, likely caused by postmortem crushing of the thin but hard eggshell. Eggshell membrane is exposed in egg number 4, just beneath the skull, and in egg number 7, just beneath the right scapula. (**b**) CT scan of a complete egg in a, showing the eggshell (es) and the detached preserved eggshell membrane (em). (**c)** Outer surface SEM image of a *Massospondylus* eggshell fragment showing rare small and irregularly shaped pores occurring in random patterns (red arrows). (**d)** Enlarged view of boxed area in (**c**). See also Figs S1–S3.

The South African *Massospondylus* (BP/1/5254, BP/1/5347) calcareous eggshell layer is slightly thicker (80–100 µm) than that of *Lufengosaurus*. The eggshell units are very difficult to discern (Fig. 1i,j). In the past, these units have been interpreted as wedge-shaped^8^. Our cathodoluminescence analysis (Figs 1k and S1) shows very high luminosity of calcite in the eggshell units, which supports the idea that these structures are the result of diagenetic alteration of the original microstructure^9^ (Figs 1k and S1). Nonetheless, eggshell is present in *Massospondylus* eggs from several different localities in South Africa, and of similar thickness as in *Lufengosaurus*, and some features remain recognizable. The outer surface of the eggshell, as in *Lufengosaurus*, is rugged with low tubercles and shallow depressions. Occasional pores are distributed unevenly throughout the shell surface (Fig. 2c,d). Below the calcareous layer, a dark, isotropic layer (50–90 µm thick, cross polarized light) merges with, or entirely obscures the mammillary cones (Fig. S3). We identify this layer as a remnant of the eggshell membrane, given its position relative to the calcareous layer and its chemical similarity with the *Lufengosaurus* shell membrane (rich in phosphate and calcite, Figs S1–S3). A shell membrane is also preserved in some of the complete eggs with the embryos (Fig. 2a).

The Argentinian *Mussaurus* eggshell (PVL 5965) is severely affected by diagenesis. Only few sparse and widely scattered calcite crystals, similar in size and shape to the radiating crystals in the mammillary cones of *Lufengosaurus* eggshell units, remain of the calcareous layer (Fig. 1g,h). The eggshell membrane is preserved as a thick (150–180 µm) phosphatic layer with little internal structure.

Calcareous layer to membrane thickness ratios may vary due to incomplete preservation of the membrane. They range from ~1:1 in *Lufengosaurus* and ~1.5:1 *Massospondylus*, but remain uncertain in *Mussaurus* due to the loss of an intact, coherent calcareous layer.

**Figure 3:**
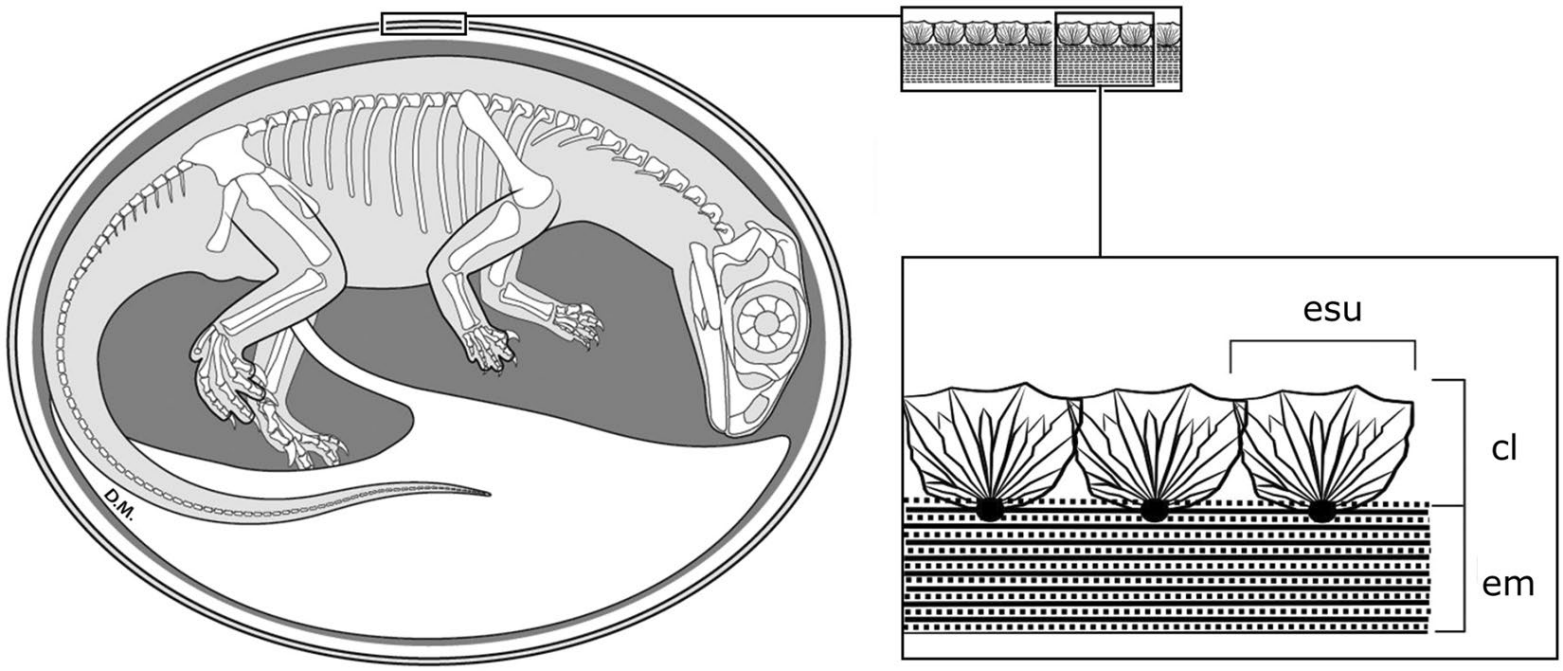
Reconstruction of a basal sauropodomorph egg showing detail of the eggshell. Eggshell units (esu) form the calcareous layer (cl) and are embedded with organic cores in the eggshell membrane (em). See also Figs S1–S3. Embryo reconstruction by R. David Mazierski with permission.

The identity of all three taxa is unquestionable based on the presence of embryonic remains^10–12,15^, *contra*^13^. We thus reconstruct basal sauropodomorph eggshell as having a thin calcareous layer, composed of low, wide mammillary cones (approximate width to height rations of 1:1) attached to a membrane of at least similar thickness (Fig. 3).

### Taphonomic and evolutionary implications

**Figure 4:**
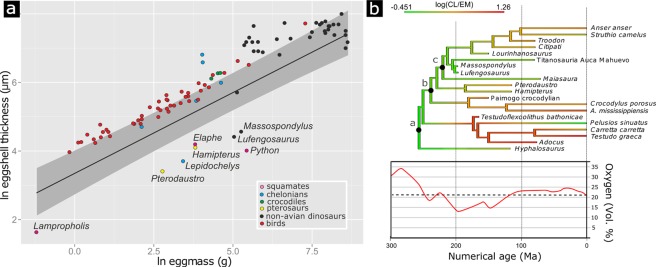
Relationship between eggshell thickness and egg mass in different egg-laying archosauromorphs and time calibrated maximum likelihood (ML) analysis of the ancestral states of relative eggshell thickness evolution. (**a**) PGLS regression line and 95% confidence band on the ln-transformed dataset. *Massospondylus* and *Lufengosaurus* represent negative outliers (see SI) emphasizing the extreme thinness of the calcareous layer compared to other dinosaurs. (**b)** ML ancestral state reconstruction of log-transformed calcareous layer thickness (CL) to egg mass (EM) ratios. Note that the root was set to represent the hypothesized ancestral flexible shelled condition. Nodes represent (**a)**, Archosauromorpha, (**b)**, Archosauria (**c)**, Ornithodira, (**d)**, Dinosauria, (**e)**, birds. Note the independent acquisitions of thick eggshell in choristoderes (represented by *Hyphalosaurus*), chelonians, crocodiles, pterosaurs and several dinosaur clades, as well as reversals in chelonians. From the Sinemurian (199 Ma) onwards, eggshells (e.g. *Testudoflexoolithus* and *Lourinhanosaurus*) show a significant calcareous layer thickness increase corresponding with atmospheric oxygen increase. See also Fig. S4.

The phylogenetically informed regression analysis of mineralized eggshell thickness versus egg mass in a wide taxonomic range of extant and extinct egg laying amniotes (Fig. 4a) revealed a significant positive relationship between egg size (mass) and shell thickness, with considerable phylogenetic signal (λ = 0.86; p < 0.001; see SI). The regression function is largely determined by the taxa with rigid-shelled eggs (non-avian dinosaurs, birds and crocodiles). Negative outliers, in which the size of the eggs and their shell thickness are well below the regression line, are the extant and fossil groups with known or inferred flexible shelled-eggs, such as marine turtles, squamates, and pterosaurs (Fig. S4). Interestingly, *Lufengosaurus* and *Massospondylus* plot with these negative outliers emphasising the pronounced thinness of their calcareous eggshell relative to their egg mass (Fig. 4). However, due to the interlocking nature of the crystal units, these basal sauropodomorphs most likely had rigid eggshell. This interpretation is supported by the preservational characteristics of all egg fragments recovered from the various sites. All retain their curvature, and even though the eggs of *Massospondylus* are somewhat crushed, they show the typical cracking and fragmenting associated with rigid structures (Fig. 1)^16,17^. This observation contrasts with the preservational characteristics of soft-shelled fossil material, now abundantly preserved for the pterosaur *Hamipterus*^18^.

A time-calibrated cladogram of archosauromorphs suggests the ancestral state for dinosaurs is the thin-shelled condition (Fig. 4b, Supplementary Information). Maximum likelihoods (ML) of ancestral character states imply low ratios of eggshell thickness to egg mass are plesiomorphic in Dinosauria. These calculations are based on the logical assumption that the archosauromorph root node represents a poorly mineralized eggshell (cf.^2,3^), the value of which is derived from the lowest observed value among the extant amniote taxa (*Pelusios sinuatus*) (see Supplementary Information). Reconstructed relative eggshell thicknesses for the base of the dinosaur tree are very close to those of the early sauropodomorphs described here. The ancestral state reconstruction suggests independent eggshell thickening events in all major archosauromorph clades, but also within different dinosaur clades. Evolutionary reversals are also demonstrated (cf. *Pelusios sinuatus* and *Carretta carretta*). Finally, it is important to note that these thickening events generally occurred after the Sinemurian (~195 Ma).

## Discussion

Our detailed examination of the eggs of these basal sauropodomorph dinosaurs shows that all have an extremely thin mineralized eggshell layer. Different diagenetic settings of their respective localities affected the original microstructure to different degrees, with *Lufengosaurus* having the best, and *Mussaurus* the least preserved details (Fig. 1, Supplementary Information). The structural characteristics of these Early Jurassic dinosaur eggshells are unlike those in any other known dinosaur. The extreme thinness could have resulted from decalcification during egg incubation, as seen in some *Massospondylus* eggs containing advanced stage embryos. However, this is unlikely because there is no sign of resorption craters at the base of the crystal units, and the recent collection of a complete *Massospondylus* nest with undeveloped embryos makes this unlikely^11^ (Fig. 2a). In addition, the similar thinness, eggshell unit characteristics, and outer surface ornamentation suggest that the calcareous shell layers are similar to their original thickness in both *Lufengosaurus* and *Massospondylus*. A low ratio of calcareous layer to eggshell membrane thickness is usually associated with flexible-shelled eggs of extant amniotes^19^, however, in line with our other observations, we conclude that these early dinosaurs had thin, albeit rigid-shelled eggs (Fig. 3), a highly unusual, unexpected condition. The semi-arid depositional conditions^7,11,12^ and relative thinness of the eggshell suggest the eggs needed to be protected from dehydration^17,20,21^. Hence, as in many other dinosaurs^22,23^ and most modern-day non-avian reptiles, the eggs were most likely buried in the nest, although this hypothesis needs further support by more complete data on pore density and relative eggshell porosity. Previous studies have pointed to a combination of nesting site fidelity, colonial nesting, and parental care in these early sauropodomorphs^11,12^. It is thus possible that through their behavioural ecology, these sauropodomorphs created taphonomic conditions that allowed the preservation of such delicate structures.

Eggshells with a comparatively thick membrane but thin calcareous layer are in sharp contrast with heavily mineralized dinosaurian eggshells commonly found during the Cretaceous^13,14^. Ancestral state reconstruction of this feature may be affected by lack of information from earlier reptilian clades and crucial basal taxa, such as early ornithischians. Nonetheless, the lack of pre-Middle Jurassic rigid fossil eggshells^3,5,24^, the different mammillary ultrastructure in crocodilian and dinosaurian eggshells, and the aragonitic nature of turtle eggshells, provide strong support for the hypothesis of independent eggshell thickening events in these reptiles (see Supplementary Information for further results and discussion of the ancestral state analysis). This scenario also favours the independent origin of extended eggshell growth in the dinosaurian clades Ornithischia, Sauropodomorpha and Theropoda. All known dinosaurian eggshells, including those described here, possess mammillae with radiating calcite crystals^14^, therefore, mammillated eggshell with calcite radial ultrastructure can be considered a dinosaurian synapomorphy.

It seems straightforward to assume a flexible non-mammillated → flexible mammillated → rigid mammillated succession of eggshell structural evolution. However, it does not have to be so strictly sequential or directional, as there may be an intricate interplay between biological and environmental factors shaping eggshell structure and composition^19^. Diversity in eggshell micro- and ultrastructure in different reptilian clades points to numerous convergences, secondary losses and reversals. Turtles demonstrate this evolutionary complexity by revealing conditional aragonite/calcite composition of the calcareous layer^19,25^ and multiple eggshell-softening events^26^ (Fig. 4b), even with complete loss of mammillae in the pleurodiran *Pelusios sinuatus*^27^.

Our ancestral state reconstruction shows an independent thickening of the calcareous layer in several archosauromorph clades during the Jurassic. Interestingly, this does not seem to be directly related to increase in body size, and hence egg size. However, the occurrence of the earliest strongly mineralized archosauromorph and turtle eggshells in the Middle and Late Jurassic^5,28^ coincides with the recovery to modern day atmospheric oxygen values (Fig. 4b)^29^. The GEOCARBSULF model suggests atmospheric oxygen levels dropped during the Permian and Triassic from an all-time high (32–33%) in the Late Carboniferous to an all-time low (15%) in the Early Jurassic^29^. Such models calculate Phanerozoic atmospheric oxygen levels by representation of nutrient cycling and estimation of productivity, or by isotope mass balance^29–31^. Estimated *p*O_2_ may vary depending on the used model, nonetheless, a negative excursion in the Hettangian (201-199 Ma) clearly precedes a general trend of atmospheric oxygen increase in the Sinemurian, 199-191 million years ago (Fig. 4b)^29,31,32^.

In modern reptiles, oxygen restriction is known to play an important inhibiting role on eggshell growth and other aspects of embryonic development^33,34^. Furthermore, *Plateosaurus*, a Norian to Rhaetian basal sauropodomorph from Central Europe and Greenland (Fig. 1) is phylogenetically close to the materials presented here and known from abundant remains (e.g.^35^), but hitherto no eggs have been found. Eggs predating the Early Jurassic would likely be very difficult to find. Nonetheless, there is no evidence of any fossil eggs preserved during the 120 million years of amniote evolution that would predate the findings described here, anywhere around the globe and in any type of depositional system. We suggest that egg physiology and low atmospheric oxygen levels may have inhibited eggshell thickening before the end of the Early Jurassic, when atmospheric oxygen levels started to rise again. However, it should be stated that this remains a hypothesis and further testing it is beyond the scope of the current study.

## Material and Methods

### Thin sectioning

The eggshell from the Early Jurassic DaWa locality in the Lower Lufeng Formation of Yunnan, China, is documented from a 3–4 cm long calcareous nodule containing numerous eggshell fragments (but no bones) (Fig. 1). The material is housed in the Chuxiong Prefectural Museum under catalogue no. C2019 2A233. Uncut shell fragments can be identified from their high Ca and P content with µXRF (Fig. S2). Radial and tangential petrographic sections were made from the sample (Figs 1a–e and S3). The eggshells were found in a 10–20 cm thick monotaxic bonebed. The layer solely contains dislocated basal sauropodomorph embryonic elements ascribed to *Lufengosaurus*^12^. In contrast to the DaWa locality, specimens from the Rooidraai locality in South Africa are complete eggs with well-preserved embryos inside (Fig. 2)^10,11,15^. Thin sectioned *Massospondylus* eggshell Figs 1i,j and S3) was not directly obtained from a nest with embryos, but sampled from an adjacent nest in the same horizon containing embryonic remains ascribed to *Massospondylus*^10^. The *Massospondylus* material is housed at the Bernard Price Institute of Palaeontology of the University of Witwatersrand under catalogue no. BP/1/5254 and BP/1/5347. Despite the taphonomical difference between Lufeng and Rooidraai, the two localities are similar in geology, temporal range, environment, and faunal assemblages^10–12^. The *Mussaurus* eggshell (Fig. 1g,h) fragment was sampled from a nest containing eggs with embryos (specimens stored at the Instituto ‘Miguel Lillo”, Tucuman, catalogue no. PVL 5965). The specimen was collected from the Early Jurassic of the Laguna Colorada Formation of Patagonia, Argentina by researchers of the Museo Paleontológico Egidio Feruglio in Trelew, near the original *Mussaurus* embryo discovery site^7^.

### Light microscopy and SEM

Fossil eggshell specimens were thin sectioned in the Steinmann Institut (University of Bonn) and studied under single plane polarizers (ppl) and cross-polarized light (xpl) under a Leica DMLP and a Zeiss Axioskop compound microscope. Photos of sections were taken with a Leica 425 firecam and Zeiss Axiocam. Scanning electron microscopy images were taken with a JEOL JSM 6300 (Tokyo, Japan).

### Cathodoluminescence

Cathodoluminescence imaging (Figs 1 and S1) was performed using a Cambridge Image Technology (CITL) Mark 5 cathodoluminescence system (Hatfield, UK) at University of Mons, Belgium. Beam conditions were 15 kV acceleration voltage and 500 μA beam current. The cold cathode electron gun produced an unfocussed elliptical beam of ca. 60 mm^2^, which results in a current density of 8 µA/mm^2^. Helium was used instead of air in order to improve beam stability. The cold cathode electron gun produced an unfocused beam of a few mm in diameter. Spectral cathodoluminescence imaging was achieved by inserting narrow bandpass optical filters within the lightpath. Filtering at 880 nm allowed observing the emission of Nd^3+^ which substitutes Ca^2+^ in apatite. In this mode, the strong yellow-red cathodoluminescence of calcite is suppressed and the infrared cathodoluminescence of apatite is enhanced. Filtering at 640 nm isolates the emission of Sm^3+^ but is also influenced by the strong cathodoluminescence of calcite, which is activated by Mn^2+^ at ca. 605 nm. The cathodoluminescence images were captured with a high-sensitivity, Peltier-cooled digital color camera. For spectral imaging, the camera was used in 2 × 2 binning mode in order to capture monochromatic images. Cathodoluminescence spectra were recorded using a CITL OSA2 optical spectrometer with a Peltier-cooled CCD detector and a spectral resolution of 4 nm. The spectra are corrected for background and ambiant light (dark measurement) but not for system response.

### µXRF and Raman spectroscopy

Identifying eggshell specimens with a thickness of only 100 to 200 µm proved sometimes equivocal under the microscope. Moreover, the identity of the membrane and calcareous shell was not always clear. Therefore, we employed spectroscopic methods to characterize chemical composition of the eggshell components. First we used µX-ray fluorescence (µXRF, M4 Tornado, Bruker Nano Technologies, Berlin, Germany) to identify major element distribution in fluorescence maps of fossil eggshell fragments (Fig. S2). Element distribution maps show a relative counts signal after deconvolution. Only elements of interest (Ca, P, Fe, Si) are highlighted. Line scans (Fig. S2c,e) show relative counts signal and were extracted from map data to demonstrate gradients of element composition along a chosen transect in the samples. µXRF results were cross referenced with Raman spectroscopy (Fig. S2f,g). We used a fully integrated confocal Raman microscope (LabRAM HR Evolution, HORIBA Scientific, Kyoto, Japan) equipped with a high stability confocal microscope with XYZ motorized stage and a multichannel air cooled CCD detector (spectral resolution <1 cm^−1^, lateral resolution 0.5 µm, axial resolution 2 µm). Two lasers are mounted on the instrument: a HeNe laser (633 nm) and a Solid state laser (532 nm). Initially, the green laser was used to reduce signal noise, but due to overheating, and even burning of the sample, the red laser had to be installed. Both lasers were used in combination with a 50x objective. Intensity for spot measurements ranged from 2.5–25 mW.

### Phylogenetic regression of eggshell thickness vs egg mass

To examine the relative rigidity of the early sauropodomorphs eggshells compared to the size of the eggs, we compiled a comprehensive dataset of calcareous eggshell thickness (mineralized calcite or aragonite layer thickness) and egg mass in a variety of fossil and extant egg-laying amniotes (two snakes, one lizard, six turtles, three crocodiles, two pterosaurs^36^, non-avian dinosaurs^37^, birds; see Table S1, Fig. 4a). Besides the early sauropodomorph eggshells measured in this study, eggshell thickness data were collected from the literature^19–24,25–28,38–43^. Egg mass data were estimated from published size data^19,20,40–44^ with the formulae M = 5.60 × 10^−4^ × L × B2 (M, egg mass; L, maximum egg length; B, maximum egg breadth) for non-avian sauropsids^19^ and M = 5.48 × 10^−4^ × L × B2 for birds^45^. Mass and dimensions of a *Lufengosaurus* egg are extremely difficult to estimate, but our values were based on a size comparison of embryonic remains with those of *Massospondylus*^10–12^. Elements belonging to *Massospondylus* embryos are generally 1.5 times smaller in length than those of *Lufengosaurus*, translating in a three times larger egg volume and mass for *Lufengosaurus*.

The egg mass and shell thickness dataset was then used in regression analyses to investigate how eggshell rigidity is reflected in the relationship between eggshell thickness and egg mass across these taxa. All calculations were performed in R version 3.2.3 (2015 The R Foundation for Statistical Computing).

To account for the trait correlations resulting from phylogenetic interrelationships on the regression outcome, a phylogenetic tree containing all taxa (nexus file S1) in the dataset was constructed in Mesquite v3.04^37^, where topologies were based on the literature^46–52^. Unknown divergence times and branch lengths were based on data of the age of the oldest fossil occurrence of eggshell taxa. For measuring phylogenetic signal in thickness of calcareous eggshell layer and egg mass, we used two different methods: Blomberg’s K, and Pagel’s λ (phylosig from package ‘phytools’^53^) both of which gave a significant phylogenetic signal for both traits (p ≤ 0.001). Relationship between eggshell thickness and egg mass was linearized by ln-transformation of both variables. Phylogenetic Generalized Least Squares (PGLS) regression (gls with Brownian motion evolution from package ‘nlme’^54^ as well as gls with Pagel’s λ scaling parameter (corPagel) from package ‘ape’^55^ was performed on the ln-transformed dataset. Based on AIC values and log-likelihoods, the λ-model fitted our data better and therefore was chosen for the interpretation of our results. 95% confidence band was visualized for the regression line (ggplot in package ‘ggplot2’^56^). Taxa with relatively thin shelled eggs were identified in the regression using three methods for outlier recognition: QQ-plot of residuals’ normality, density plot of residuals, and boxplot.stats function (Fig. S4).

### Ancestral state reconstruction of eggshell features

After identifying the most likely physical properties of fossil eggshells by means of phylogenetic regression, we focused on known fossil archosauromorph eggshells, and computed the ancestral states of the ratio of calcareous layer thickness to egg mass in a variety of taxa. Only limited fossil specimens were available for this analysis, and were balanced with extant species of all known modern archosauromorph clades. Taxa and values are listed in Table S2.

Tree topology (nexus file S2) was compiled from literature data. The position of pterosaurs is based on^52^ and^57^ (but see^58,59^ for contrasting views). Choristoderes are placed as basal archosauromorphs^60,61^, and turtles are considered sister taxon to archosaurs, based on current molecular evidence^47,62–64^, but see^65^ for a contrasting view). Divergence dates were collected from literature data^46–52,57^ and the Paleobiology Database.

Reconstruction of ancestral states was computed in R using the functions ‘fastAnc’ and ‘contMap’ of the phytools package^53^, with the root node set to represent the ancestral poorly mineralized eggshell, a hypothetical value based on 2 and 3, and the lowest observed value among the extant taxa in our analysis (*Pelusios sinuatus*). The value for the root was set at 1.05 because it maximizes observable differences between reconstructed and observed states.

## Supplementary information

Supplementary information with text, figures and tables

Supplementary Dataset 1

Supplementary Dataset 2

## Data Availability

The datasets generated during and/or analysed during the current study are available from the corresponding authors on reasonable request.
