# Supplementary material for: Structure and evolutionary implications of the earliest (Sinemurian, Early Jurassic) dinosaur eggs and eggshells
Source: Sci Rep. 2019 Mar 14;9:4424. doi: 10.1038/s41598-019-40604-8 (PMC6418122; doi:10.1038/s41598-019-40604-8)
Supplement: Supplementary file 1 — Supplementary information with text, figures and tables [file 41598_2019_40604_MOESM1_ESM.docx]

**Supplementary Information for**

**Structure and evolutionary implications of the earliest (Sinemurian, Early Jurassic) dinosaur eggs and eggshells**

**Authors:** Koen Stein^1,2*°^, Edina Prondvai^3,4^, Timothy Huang^5,6^, Jean-Marc Baele^7^, P. Martin Sander^8,9^, Robert Reisz^5,6,10*^

**Affiliations:**

^1^Earth System Science - AMGC, Vrije Universiteit Brussel, Pleinlaan 2, 1050 Brussels, Belgium.

^2^Royal Belgian Institute of Natural Sciences, Directorate 'Earth and History of Life', Rue Vautier 29, 1000 Brussels, Belgium.

^3^Evolutionary Morphology of Vertebrates, Ghent University, K.L. Ledeganckstraat 35, 9000 Gent, Belgium

^4^MTA-ELTE Lendület Dinosaur Research Group, Eötvös Loránd University, Pázmány P. s. 1/C, 1117 Budapest, Hungary.

^5^ International Center of Future Science, and Dinosaur Evolution Research Center of Jilin University, Changchun, Jilin Province, China

^6^ National Chung Hsing University, Taichung 402, Taiwan.

^7^ Department of Geology and Applied Geology, Faculty of Engineering, University of Mons, Place du Parc 20, 7000 Mons, Belgium

^8^ Steinmann Institute of Geology, Mineralogy, and Paleontology, Division of Paleontology, University of Bonn, Nussallee 8, 53115 Bonn, Germany.

^9^ Natural History Museum of Los Angeles County, Dinosaur Institute, 900 Exposition Boulevard, Los Angeles, CA 90007, USA

^10^ Department of Biology, University of Toronto Mississauga, Mississauga, Ontario L5L 1C6, Canada.

* Correspondence to: kstein@vub.be; robert.reisz@utoronto.ca

° Lead Contact

Supplementary Text

*Preservation and geochemistry of the basal sauropodomorph eggshell*

Eggshell membrane remains have previously been reported in fossil eggshells (e.g. 41, 67-69); however, their presence in Early Jurassic dinosaur eggshells is unprecedented. *In vivo* eggshell membrane is largely composed of protein fibers similar to collagen type I as well as proteoglycans (70), and has several functions including water balance, mechanical support and protection against microbes (20, 71). The structures which we identified as fossil eggshell membranes mostly contain apatite (Supplementary Fig. S1-S3). Recently, eggshell membrane of the hen was used as a medium to artificially grow flower-like clusters of apatite minerals (72, 73), similar to those observed in the *Lufengosaurus* material (Supplementary Fig. S4). Francolite, a carbonate rich morph of hydroxylapatite, has also been shown to precipitate in organic matter-rich carbonates under the anoxic conditions created in lagoonal bacterial mats (74). It is thus more than likely that anoxic conditions prevailed during early diagenesis, and the basal sauropodomorph egg membrane served as a medium for the precipitation of calcium phosphates derived from the decaying embryos. Over time, the apatite replaced the membranous proteins entirely, and the phosphatic nature allowed the long term preservation of these structures. Such membrane phosphatization processes may also explain the preservation of other fossil egg membranes (2, 41, 75, 76).

*Extended results of ancestral states and the evolution of a thickened eggshell*

The structural similarities (mammillary cones, radiating to fan-shaped carbonate ultrastructure of crystal units) of the eggshell of *Lufengosaurus* with that of crocodiles and marine turtles (cf. 20, 25, 26, 77), and the accumulating evidence that turtles are sister group to archosaurs (48, 63-65, but see 66 for an opposing view), may suggest that a simple fan-shaped extension of the calcareous mammillary cones by radial and appositional growth, i.e. the spherulitic morphotype, is plesiomorphic for Archosauromorpha. This suggestion also implies that a mammillated eggshell is homologous in turtles, crocodiles, and dinosaurs. However, there are multiple questions and problems related to this hypothesis implying an independent acquisition of a hard calcareous eggshell layer in these archosauromorph clades (cf. 15). Firstly, there is no known well-mineralized eggshell from earlier than Middle Jurassic (3, 5, 27, 29) by which time these three major archosauromorph clades had already diverged and thus underwent independent evolution. Second, the following archosauromorph clades show deviations from what would be expected in case of a single homologous origin of the rigid mammillated eggshell or exhibit peculiarities not fitting in the derived archosauromorph condition:

1. *Crocodiles.* Unlike in *Lufengosaurus* and other dinosaur eggshells, mammillary processes in crocodilians seem to lack the typical calcite radial ultrastructure (8) but instead they show rosette-like structures composed of platy crystallite aggregates (39). Whether this is a primary or a secondary feature in crocodiles is hard to judge because nothing is known about the eggs of the earliest crocodylomorphs. Either way, it is highly likely that ancestral crocodylomorphs also had a calcitic eggshell with units composed of mammillary processes with or without a radial ultrastructure and additional layers. However, the relative thickness of this calcitic layer, and hence the flexibility properties of the eggshell could have varied in extinct crocodylomorphs, as it does in modern turtles. Furthermore, some highly specialized crocodyliforms, such as marine thalattosuchians with aquatic adaptations may still have had calcareous eggs and a nesting strategy similar to modern marine turtles; however, more derived hydropelvic thalattosuchians could have evolved viviparity, similar to most secondarily aquatic sauropsids such as mesosaurs (78), ichthyosaurs (3, 79), and mosasaurs (80).
2. *Turtles*. Even though turtles show a typical radial eggshell ultrastructure, this is formed of aragonite as opposed to the calcitic eggshell of crocodiles, dinosaurs, and even squamates. However, in seawater aragonite typically precipitates in preference of calcite under high Mg/Ca conditions. The calcium carbonate morph of the calcareous shell may thus be influenced by nutritional and/or environmental factors (20): captive sea turtles that usually produce an aragonitic eggshell have been shown to lay eggs containing both calcium carbonate morphs (26), and eggshells of some captive pythons may be aragonitic rather than calcitic (81). The aragonitic nature of turtle eggshell therefore does not exclude a homologous origin of the calcareous layer in Archosauromorpha but rather seems to be a secondary modification in the turtle lineage.
3. *Pterosaurs*. Interestingly, pterosaurs, widely regarded as sister group to dinosauromorphs (53), also have an extremely thin eggshell. Initial observations on pterosaur eggshell claimed structural similarities with dinosaur eggshell (82), but subsequent reports suggested they do not possess a mammillary layer, nor does their eggshell share any structural similarities with crocodiles or other archosaurs (43, 4483-85). Instead, pterosaurs show strong structural similarities with the parchment-like squamate eggshells (20, 43, 44). It is unlikely that the absence of a distinct mammillary layer in pterosaurs is a diagenetic artifact (44). This feature may thus add to the alternative hypothesis that pterosaur ancestors have diverged from the archosaurian lineage at a much earlier stage than commonly assumed (cf. 59, 60).
4. *Choristoderes.* Choristoderes, a group of diapsid reptiles that classically has been difficult to place phylogenetically (61, 62, 86), also show a flexible eggshell with a microstructure similar to parchment-like squamate eggshell (87). Choristoderes have been placed among archosauromorphs (61, 62); however, the lack of organic cores and mammillary knobs in their poorly mineralized calcareous shell favors the view of a more basal position among diapsids (85).

The origin and evolution of the calcareous eggshell within Dinosauria seems somewhat less confusing. Parataxonomic descriptions of dinosaur eggshells show that all dinosauroid and ornithoid structural morphotypes have mammillary cones with an organic core and radiating calcite crystallites which in life was embedded in the ESM (14). This character is considered here a synapomorphy of Dinosauria. In the derived ornithischian *Maiasaura* (88) and possibly *Telmatosaurus* (89), as well as in the saurischian sauropods and basal theropod *Torvosaurus* (4, 5), the thick spherulitic eggshell units can be explained by a continued crystal growth of the mammillary cones into fan-shaped or even branching structures. This thickening mechanism of the dinosaurian spherulitic eggshell units may have evolved already in the basalmost dinosaurs or alternatively could have appeared independently in Ornithischia, Sauropodomorpha and Theropoda. The reconstructed ancestral state at the Sauropodomorpha – Theropoda node being thin, likely flexible eggshell, and the complete lack of rigid dinosaurian eggshells from earlier than the Middle Jurassic period favor the latter hypothesis.

In most other non-avian and avian theropods, the distinct border between the mammillary cones with radiating crystallites, and the palisade layer with structurally different organization and occasionally multiple layers is most probably modulated through specialized physicochemical mechanisms and specific organic molecules, just like in birds (90, 91). Therefore, this structural characteristic is clearly a derived condition in advanced theropods and probably represents a synapomorphic feature of this clade.

**References**

67. Kohring, R. Calcified shell membranes in fossil vertebrate eggshell: Evidence for preburial diagenesis. *J. Vertebr. Paleontol.* **19**, 723–727 (1999).

68. Grellet-Tinner, G. Membrana testacea of titanosaurid dinosaur eggs from Auca Mahuevo (Argentina): Implications for exceptional preservation of soft tissue in Lagerstätten. *J. Vertebr. Paleontol.* **25**, 99–106; [10.1671/0272-4634(2005)025[0099:mtotde]2.0.co;2](http://doi.org/10.1671/0272-4634(2005)025%5B0099:mtotde%5D2.0.co;2) (2005).

69. Schweitzer, M. H., Chiappe, L. M., Garrido, A. C., Lowenstein, J.M. & Pincus, S. H. Molecular preservation in Late Cretaceous sauropod dinosaur eggshells. *P. Roy. Soc. B: Biol. Sci.* **272**, 775–784; [10.1098/rspb.2004.2876](http://doi.org/10.1098/rspb.2004.2876) (2005).

70. Wong, M., Hendrix, M. J. C., von der Mark, K., Little, C. & Stern, R. Collagen in the egg shell membranes of the hen. *Dev. Biol.* **104**, 28–36; [10.1016/0012-1606(84)90033-2](http://doi.org/10.1016/0012-1606(84)90033-2) (1984).

71. Bellairs, R. & Boyde, A. Scanning electron microscopy of the shell membranes of the hen's egg. *Z. Zellforsch. Mik. Ana.* **96**, 237–249; [10.1007/BF00338771](http://doi.org/10.1007/BF00338771) (1969).

72. Li, N. *et al.* Subtleties of biomineralisation revealed by manipulation of the eggshell membrane. *Biomaterials* **32**, 8743–8752; 10.1016/j.biomaterials.2011.08.007 (2011).

73. Zhang, Y., Liu, Y., Ji, X., Banks, C. E. & Song, J. Flower-like agglomerates of hydroxyapatite crystals formed on an egg-shell membrane. *Colloid. Surface. B*  **82**, 490–496; 10.1016/j.colsurfb.2010.10.006 (2011)

74. Tribovillard, N., Récourt, P. & Trentesaux, A. Bacterial calcification as a possible trigger for francolite precipitation under sulfidic conditions. *C. R. Geosci.* **342**, 27–35[; 10.1016/j.crte.2009.10.007](http://doi.org/10.1016/j.crte.2009.10.007) (2010).

75. Xiao, S., Zhang, Y. & Knoll, A.H. Three-dimensional preservation of algae and animal embryos in a Neoproterozoic phosphorite. *Nature* **391**, 553–558 (1998).

76. Steiner, M., Zhu, M., Li, G., Qian, Y. & Erdtmann, B.-D. New Early Cambrian bilaterian embryos and larvae from China. *Geology* **32**, 833–5; [10.1130/G20567.1](http://doi.org/10.1130/G20567.1) (2004).

77. Mikhailov, K.E. Classification of fossil eggshells of amniotic vertebrates. *Acta Palaeontol. Pol.* **36**, 193–238 (1991).

78. Piñeiro, G., Ferigolo, J., Meneghel, M. & Laurin, M. The oldest known amniotic embryos suggest viviparity in mesosaurs. *Hist. Biol.* **24**, 620–630; [10.1080/08912963.2012.662230](http://doi.org/10.1080/08912963.2012.662230) (2012).

79. Sander, P. M. A fossil reptile embryo from the Middle Triassic of the Alps. *Science* **239**, 780 (1988).

80. Caldwell, M. W. & Lee, M. S. Y. Live birth in Cretaceous marine lizards (mosasauroids). *P. Roy. Soc. B: Biol. Sci.* **268,** 2397–2401; [10.1098/rspb.2001.1796](http://doi.org/10.1098/rspb.2001.1796) (2001).

81. Solomon, S. E. & Reid, J. The effect of the mammillary layer on eggshell formation in reptiles. *Animal Technol.* **34**, 1-10 (1983).

82. Chiappe, L.M., Codorniú, L., Grellet-tinner, G. & Rivarola. D. Palaeobiology: Argentinian unhatched pterosaur fossil. *Nature* **432,** 571 (2004).

83. Ji, Q.*et al.* Pterosaur egg with a leathery shell. *Nature* **432**, 572 (2004).

84.Lu, J. *et al*. An Egg-Adult Association, Gender, and Reproduction in Pterosaurs. *Science* **331**, 321–324; [10.1126/science.1197323](http://doi.org/10.1126/science.1197323) (2011).

85. Grellet-Tinner, G. *et al.*. The first pterosaur 3-D egg: Implications for *Pterodaustro guinazui* nesting strategies, an Albian filter feeder pterosaur from central Argentina. *Geosci. Front.* **5**, 759-765 (2014).

86. Gao, K. Q. & Fox, R. C. New choristoderes (Reptilia: Diapsida) from the Upper Cretaceous and Palaeocene, Alberta and Saskatchewan, Canada, and phylogenetic relationships of Choristodera. Zool. J. Lin. Soc. **124,** 303–353 (1998).

87. Hou, L. H., Li, P. P., Ksepka, D. T., Gao & K. Q., Norell, M. A. Implications of flexible-shelled eggs in a Cretaceous choristoderan reptile. *P. Roy. Soc. B: Biol. Sci.* **277**, 1235–1239; [10.1098/rspb.2009.2035](http://doi.org/10.1098/rspb.2009.2035) (2010).

88. Horner, J. R. & Makela, R. Nest of juveniles provides evidence of family structure among dinosaurs. *Nature* **282**, 296–298 (1979).

89. Grigorescu, D., Garcia, G., Csiki, Z., Codrea, V. & Bojar, A.-V. Uppermost Cretaceous megaloolithid eggs from the Haţeg Basin, Romania, associated with hadrosaur hatchlings: Search for explanation. *Palaeogeogr., Palaeocl.*  **293**, 360–374; [10.1016/j.palaeo.2010.03.031](http://doi.org/10.1016/j.palaeo.2010.03.031) (2010).

90. Nys, Y., Gautron, J., Garcia-Ruiz, J. M. & Hincke, M. T. Avian eggshell mineralization: biochemical and functional characterization of matrix proteins. *C. R. Palévol* **3**, 549–562; [10.1016/j.crpv.2004.08.002](http://doi.org/10.1016/j.crpv.2004.08.002) (2004).

91.Hernández-Hernández, A., Vidal, M. L., Gómez-Morales, J., Rodríguez-Navarro, A.B., & Labas, V. Influence of eggshell matrix proteins on the precipitation of calcium carbonate. *J. Cryst. Growth* **310**, 1754-1759 (2008).

**
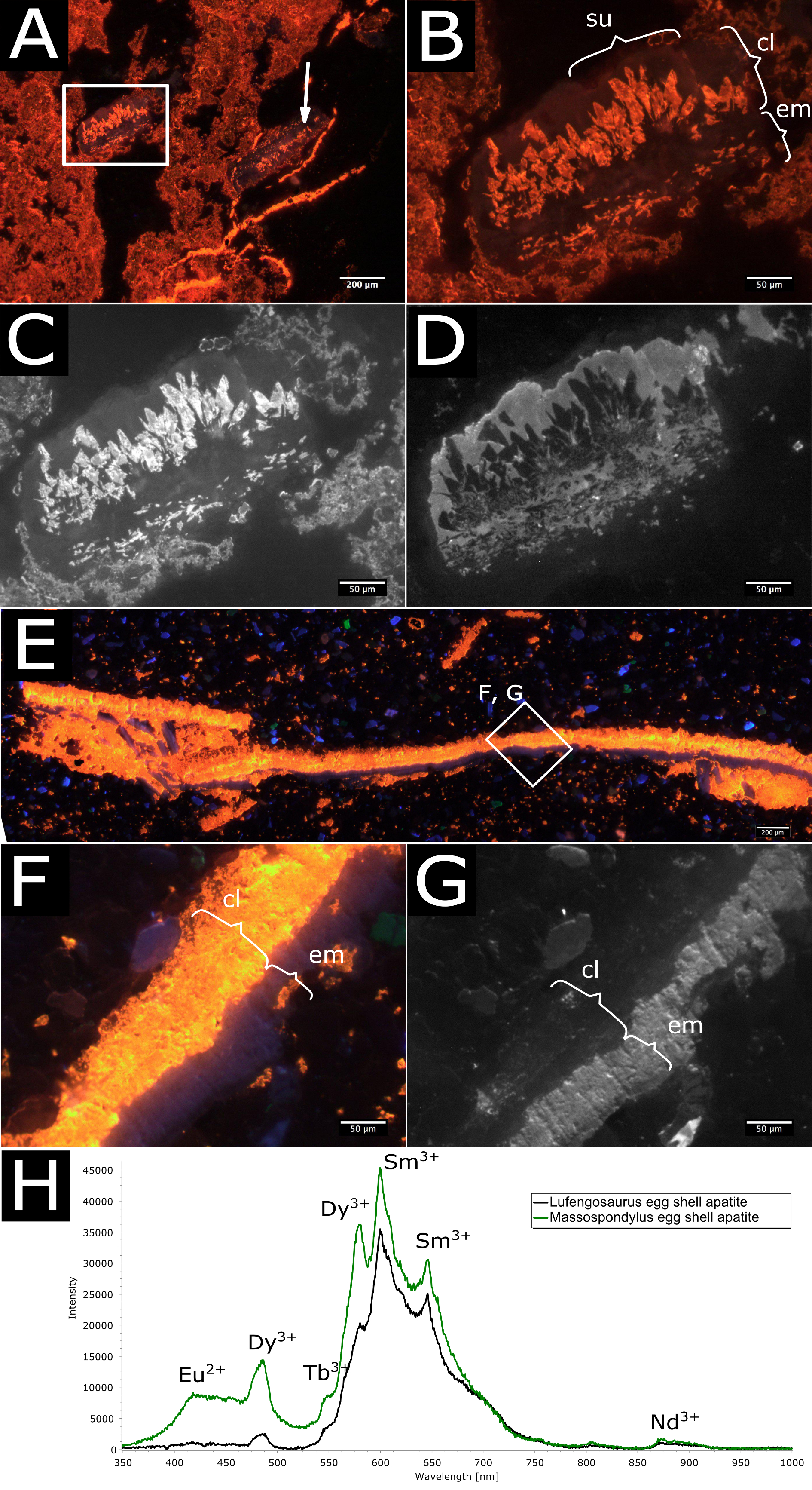
**

**Figure S1. Cathodoluminescence analysis**

A-D Cathodoluminescence images of *Lufengosaurus* eggshell. A, B, images with no spectral filter. Boxed area and arrow represent eggshell fragments. B, magnified boxed area in A, with a calcareous layer (cl) showing some signs of recrystallization of the eggshell unit (su) wedges, but overall conservation of the microstructure. C and D, same image as b with 640 nm and 880 nm filter respectively. In c, the cathodoluminescence of Sm^3+^ in apatite of the cl is obscured by the intense emission of calcite. Bright areas in the calcareous layer and membrane in D are indicative of Nd^3+^-activated apatite, in which the microstructure is more detailed, especially the fan-shape structure of the shell units. E-G: Cathodoluminescence images of *Massospondylus* eggshell. E, F, images without spectral filter. The apatite here has a distinctive blueish emission colour, which could be assigned to Eu^2+^ (D). B, magnified boxed area in A, with a calcareous layer (cl) showing intense luminescence indicative of total recrystallization. G, same image as F with 880 nm filter. Bright areas in the eggshell membrane (em) are indicative of apatite with rare earth element contents (e.g. Nd^3+^). H, cathodoluminescence spectra of *Lufengosaurus* and *Massospondylus* eggshell, showing rare earth element activation, typical of apatite (Eu^2+^, Tb^3+^, Dy^3+^, Sm^3+^ and Nd^3+^). The spectra may include some of the intense, broad luminescence of nearby calcite. Differences in the spectra show Eu^2+^ and a more intense Dy^3+^ emission in *Massospondylus*, which points to different diagenetic conditions, as these rare earth elements migrate into the apatite structure during diagenesis.


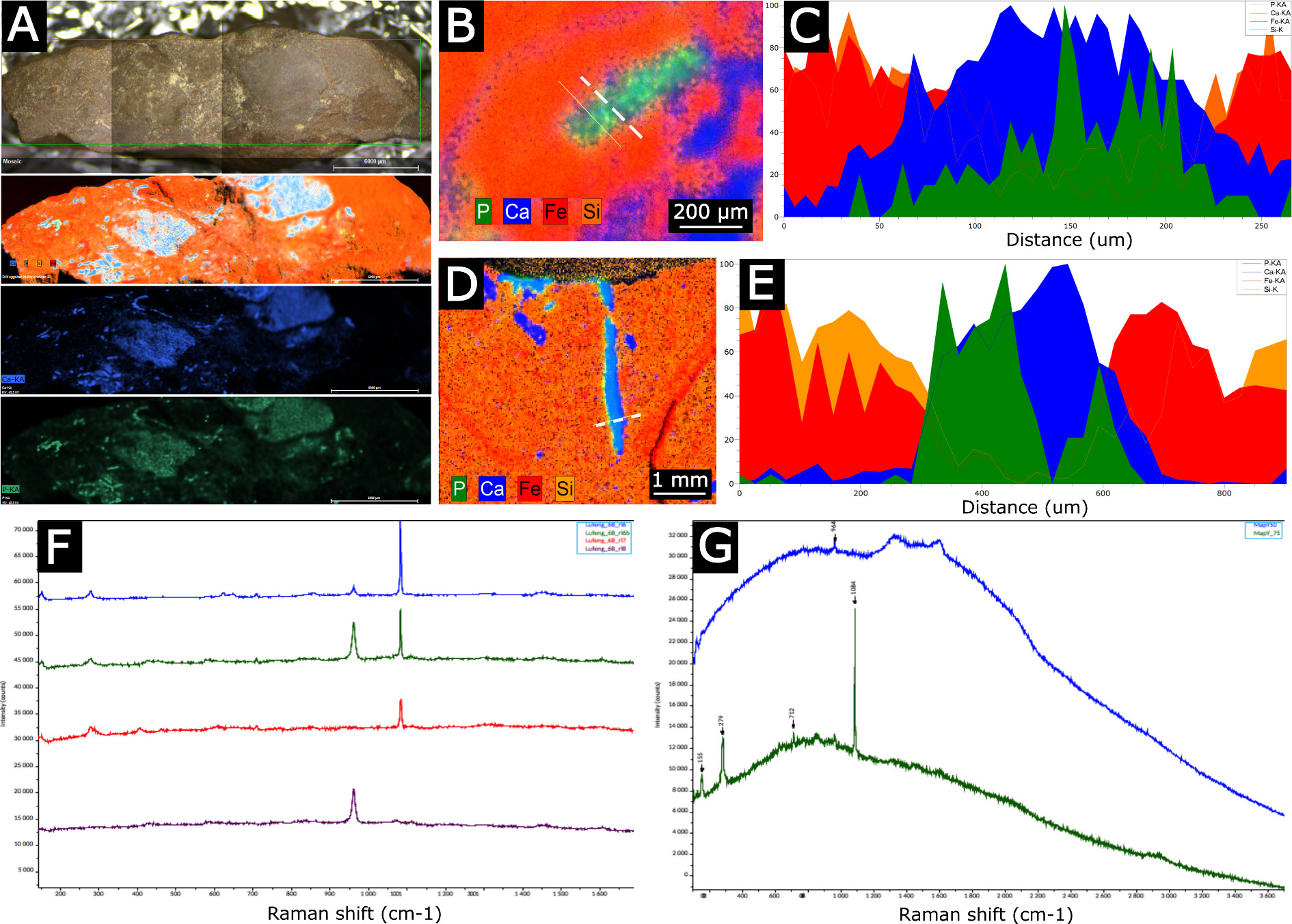


**Figure S2 µXRF and Raman analysis**

A-E µXRF maps and spectra of *Lufengosaurus* (A-C) and *Massospondylus* (D-E) eggshell. a, *Lufengosaurus* eggshell fragments identified with µXRF. A video image of the specimen shows smoother areas suspected to be eggshell. Combined element µXRF maps clearly show matrix (Si, orange, Fe, red), distinct from eggshell (Ca, blue and P, green). The map of Ca, showing the extent of the calcareous layer of the eggshell fragment, whereas a map of P, shows the extent of the preserved eggshell membrane. B, D, µXRF map showing high relative abundance of Ca and P in both eggshells. C, E, µXRF line scan along dashed line in b and d respectively, showing relative abundances (%) of Ca and P along the transect. Ca peaks correspond to the calcareous layer, P peaks to the eggshell membrane. F, Raman spectra for point measurements in *Lufengosaurus* eggshell. Different color lines show point measurements in different areas of the eggshell. A strong calcite signal (red line, peaks at 155, 280, 710 and 1084 cm^-1^) in the calcareous part of the eggshell, phosphate (purple line, peak at 964 cm^-1^) in the membrane, and mixed calcite and phosphate signals at their respective boundary (blue and green) support XRF results. G, Raman spectra for *Massospondylus* eggshell show a strong calcite signal (green line, peaks at 155, 280, 710 and 1084 cm^-1^) in the calcareous part of the eggshell, and a moderate phosphate signal (blue line, peak at 961 cm^-1^) in the membrane. Amorphous carbon detected at 1350 and 1600 cm^-1^ is probably the result of laser ablation due to too long exposure time.


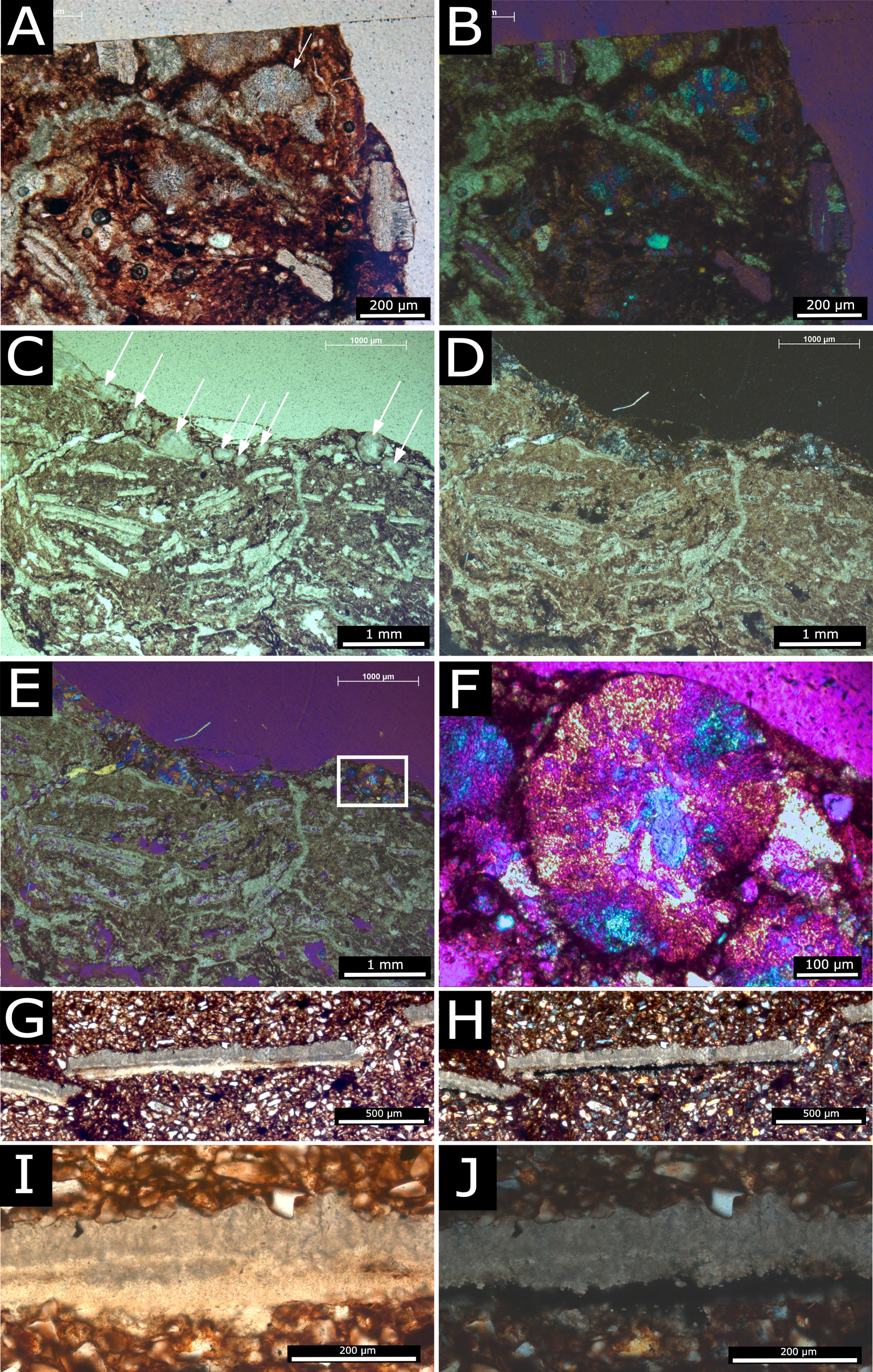


Figure S3. Polarized light microscopy

A-F Microscope images of *Lufengosaurus* eggshell, including tangential section showing rounded flowerlike cluster arrangements of crystallites (indicated with white arrows). A, specimen showing eggshell fragments and at least one rounded flowerlike cluster (white arrow). Plane polarized light. B, same as a under cross polarized light with lambda waveplate. C, plane polarized light showing multiple rounded clusters (white arrows) and radially cut eggshell fragments. D, same as c under cross polarized light. E, same as D with lambda waveplate. F, magnification of boxed area in E, detailing a rounded cluster of crystallites. G-J *Massospondylus* eggshell. G, under plane polarized light. H, under cross polarized light. I, magnification of G. J, magnification of H.


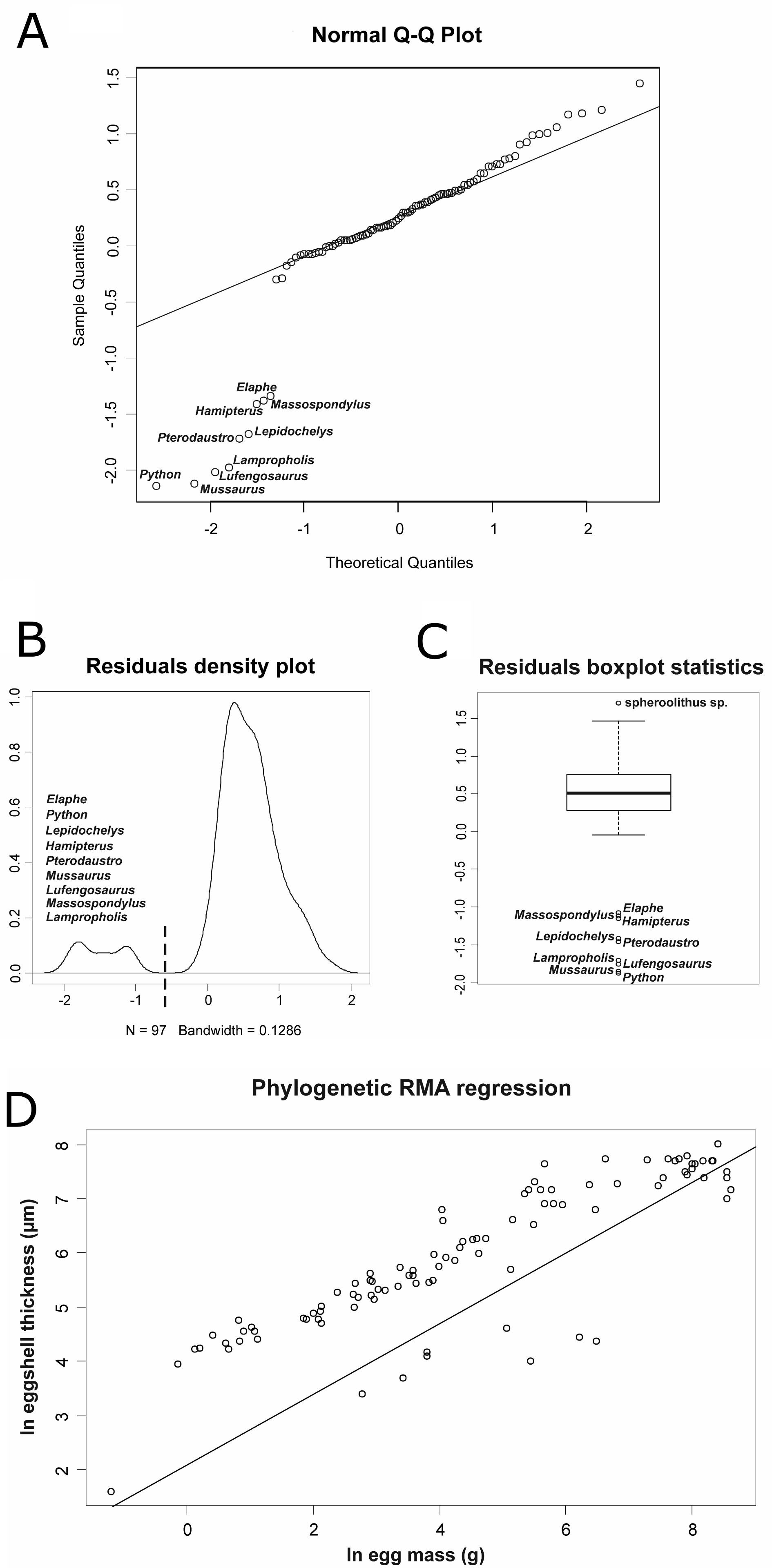


Figure S4 Pgls regression statistics.

A-C Plots of the three outlier detection techniques applied on the PGLS regression. A, QQ-plot of residuals’ normality, B, density plot of residuals, and C, boxplot of residuals recognizing the same 9 data points as regression outliers. D, Phylogenetic Reduced Major Axis Regression performed on the eggshell thickness vs. egg mass dataset. The trajectory of the regression line is largely void of measured values indicating that this method has a poor prediction and outlier recognition power on this dataset.

Table S1. Eggshell thickness and egg mass data used in the Phylogenetic Generalized Least Squares Regression. Related to Figure 4 and S4

| **Taxon** | **Eggshell thickness (µm)** | **Egg mass (g)** |
| --- | --- | --- |
| *Prinia_gracilis* | 69 | 1.12 |
| *Carduelis_carduelis* | 70 | 1.23 |
| *Passer_moabiticus* | 88 | 1.5 |
| *Muscicapa_striata* | 76 | 1.85 |
| *Carduelis_chloris* | 68 | 1.94 |
| *Melopsittacus_undulatus* | 116 | 2.25 |
| *Erythropygia_galactotes* | 80 | 2.3 |
| *Lanius_nubicus* | 95 | 2.46 |
| *Passer_domesticus* | 102 | 2.76 |
| *Galerida_cristata* | 95 | 2.93 |
| *Pycnonotus_capensis* | 83 | 3.05 |
| *Turdus_merula* | 122 | 6.36 |
| *Streptopelia_senegalensis* | 120 | 6.63 |
| *Streptopelia_decaocto* | 132 | 7.45 |
| *Streptopelia_risoria* | 119 | 8.03 |
| *Streptopelia_turtur* | 137 | 8.3 |
| *Glareola_pratincola* | 151 | 8.42 |
| *Falco_naumanni* | 194 | 10.84 |
| *Athene_noctua* | 187 | 14 |
| *Chlidonias_leucoptera* | 148 | 14.04 |
| *Gallinula_chloropus* | 229 | 14.29 |
| *Corvus_corone* | 177 | 14.99 |
| *Falco_tinnunculus* | 242 | 18.09 |
| *Alectoris_graeca* | 278 | 18.23 |
| *Himantopus_himantopus* | 185 | 18.51 |
| *Tyto_alba* | 241 | 18.71 |
| *Sterna_hirundo* | 171 | 19.5 |
| *Nycticorax_nycticorax* | 205 | 20.75 |
| *Bubulcus_ibis* | 204 | 23.22 |
| *Egretta_garzetta* | 218 | 28.52 |
| *Phasianus_colchicus* | 308 | 29.22 |
| *Burhinus_oedicnemus* | 266 | 33.51 |
| *Gallus_domesticus* | 295 | 35.88 |
| *Strix_aluco* | 268 | 36.14 |
| *Larus_ridibundus* | 231 | 37.5 |
| *Ardea_cinerea* | 242 | 49.6 |
| *Geronticus_eremita* | 394 | 50.16 |
| *Anas_platyrhynchos* | 315 | 54 |
| *Buteo_rufinus* | 371 | 60.72 |
| *Bubo_bubo* | 349 | 69.3 |
| *Ciconia_ciconia* | 502 | 78.78 |
| *Aquila_rapax* | 520 | 92.83 |
| *Anser_anser* | 741 | 173.01 |
| *Gyps_fulvus* | 676 | 243.88 |
| *Nectarinia_osea* | 52 | 0.86 |
| *Struthio_camelus* | 2245 | 1460.85 |
| *Alligator* | 530 | 99.35 |
| *Crocodylus_niloticus* | 530 | 113.61 |
| *Crocodylus_acutus* | 450 | 75.08 |
| *Lampropholis guichenoti* | 5 | 0.3 |
| *Python_molurus* | 55 | 230 |
| *Elaphe* | 65 | 45 |
| *Lepidochelys_kempi* | 40 | 30.73 |
| *Geochelone_elephantopus* | 400 | 101.64 |
| *Chelydra_serpentina* | 110 | 8.34 |
| BMNH_47208 | 900 | 56.7 |
| *Testudoolithus_jiangi* | 730 | 57.7 |
| *Testudoolithus_rigidus* | 235 | 46.43 |
| Elongatoolithidae | 1300 | 323 |
| *Macroolithus_yaotunensis* | 1450 | 911 |
| *Macroolithus_rugustus* | 1420 | 592 |
| *Elongatoolithus_andrewsi* | 980 | 384 |
| *Lourinhanosaurus_antunesi* | 900 | 643 |
| *Macroelongatoolithus_xixianensis* | 2100 | 3145 |
| *Prismatoolithus_levis*_troodontid | 1000 | 331 |
| indet_theropod1 | 300 | 168 |
| indet_theropod2 | 1300 | 224 |
| indet_theropod3 | 1200 | 210 |
| indet_theropod4 | 1300 | 273 |
| Titanosaur | 2300 | 2045 |
| Titanosaur_Type2 | 1400 | 1756 |
| *Cairanoolithus_roussetenssis* | 1700 | 2751 |
| *Cairanoolithus_dughii* | 2100 | 3001 |
| *Cairanoolithus_dughii*_Titanosaur_Type1 | 1900 | 3001 |
| *Megaloolithus*_sp.1 | 1600 | 1890 |
| *Megaloolithus*_sp.2 | 2400 | 2751 |
| *Megaloolithus_mamillare*_Titanosaur_type3.1 | 1600 | 5186 |
| *Megaloolithus_mamillare*_Titanosaur_type3.2 | 1300 | 5565 |
| *Megaloolithus_mamillare*2 | 1800 | 5186 |
| *Megaloolithus_siruguei_*Titanosaur_Type4_1 | 2200 | 3546 |
| *Megaloolithus_siruguei*_Titanosaur_Type4_2 | 2200 | 4121 |
| *Megaloolithus_siruguei1* | 3000 | 4536 |
| *Megaloolithus_siruguei3* | 2200 | 4152 |
| *Megaloolithus_sp._Telmatosaurus_transsylvanicus* | 2300 | 2446 |
| *Megaloolithus_aureliensis2* | 1100 | 5186 |
| *Megaloolithus_pseudomamillare* | 1600 | 3629 |
| *Megaloolithus_petralta* | 1800 | 2691 |
| *Megaloolithus_microtuberculata* | 2200 | 2294 |
| *Massospondylus* | 100 | 158 |
| *Mussaurus* | 80 | 660 |
| *Lufengosaurus* | 85 | 500 |
| spheroolithidae | 1500 | 247 |
| *Spheroolithus_albertensis* | 1000 | 287 |
| *Spheroolithus_sp.* | 2100 | 287 |
| Dendroolithidae | 2300 | 751 |
| MHIN_UNSL_GEO_V246_*Pterodaustro* | 30 | 16 |
| *Hamipterus_tianshanensis* | 60 | 45 |

Table S2. Relative values of eggshell thickness versus egg mass data used in the ancestral state reconstruction. Taxa are arranged in ascending egg mass value. Note how *Lufengosaurus* and *Massospondylus* have a thin shell compared to a comparably sized goose (*Anser*) and crocodile (*Crocodylus*) egg. Related to Figure 4 and S4.

| **taxon** | **Shell thickness (µm)** | **Egg mass (g)** |
| --- | --- | --- |
| *Hyphalosaurus_baitaigouensis* | 3 | 2,085 |
| *Pelusios_sinuatus* | 2,9 | 8,183 |
| *Testudo_graeca* | 270 | 14,838 |
| *Pterodaustro* | 50 | 19,354 |
| *Adocus* | 529 | 38,596 |
| *Hamipterus* | 60 | 40,136 |
| *Caretta_caretta* | 84 | 41,489 |
| *Mussaurus_patagonicus* | ? | 68,040 |
| *Alligator_mississippiensis* | 510 | 69,055 |
| *Crocodylus_porosus* | 300 | 116,596 |
| *Lufengosaurus_huenei* | 75 | 153,790 |
| *Anser_anser* | 530 | 156,695 |
| *Massospondylus_carinatus* | 120 | 192,080 |
| *Troodon_formosus* | 1000 | 339,476 |
| *Citipati_osmolskae* | 1000 | 483,336 |
| *Lourinhanosaurus* | 900 | 784 |
| *Maiasaura* | 972 | 967,68 |
| Titanosauridae (Auca Mahuevo) | 1244 | 1225 |
| *Struthio_camelus* | 2000 | 1389,18 |
